# Metapneumovirus-associated necrotizing disseminated acute leukoencephalopathy

**DOI:** 10.1055/s-0046-1820527

**Published:** 2026-05-18

**Authors:** Sara Caixeta de Souza, Ahmed Haydar, João Lucas Silva Santana, Artur Martins De March, Guilherme Diogo Silva, Fernando Freua, Luis Filipe de Souza Godoy, Leandro Tavares Lucato, Adalberto Studart Neto, Rodrigo Holanda Mendonça, Eduardo Genaro Mutarelli, Marcia Rubia Rodrigues Gonçalves, Ida Fortini

**Affiliations:** 1 Universidade de São Paulo, Faculdade de Medicina, Hospital das Clínicas, Divisão de Neurologia, São Paulo SP, Brazil.; 2 Universidade de São Paulo, Faculdade de Medicina, Hospital das Clínicas, Divisão de Radiologia, São Paulo SP, Brazil.

**Figure 1:**
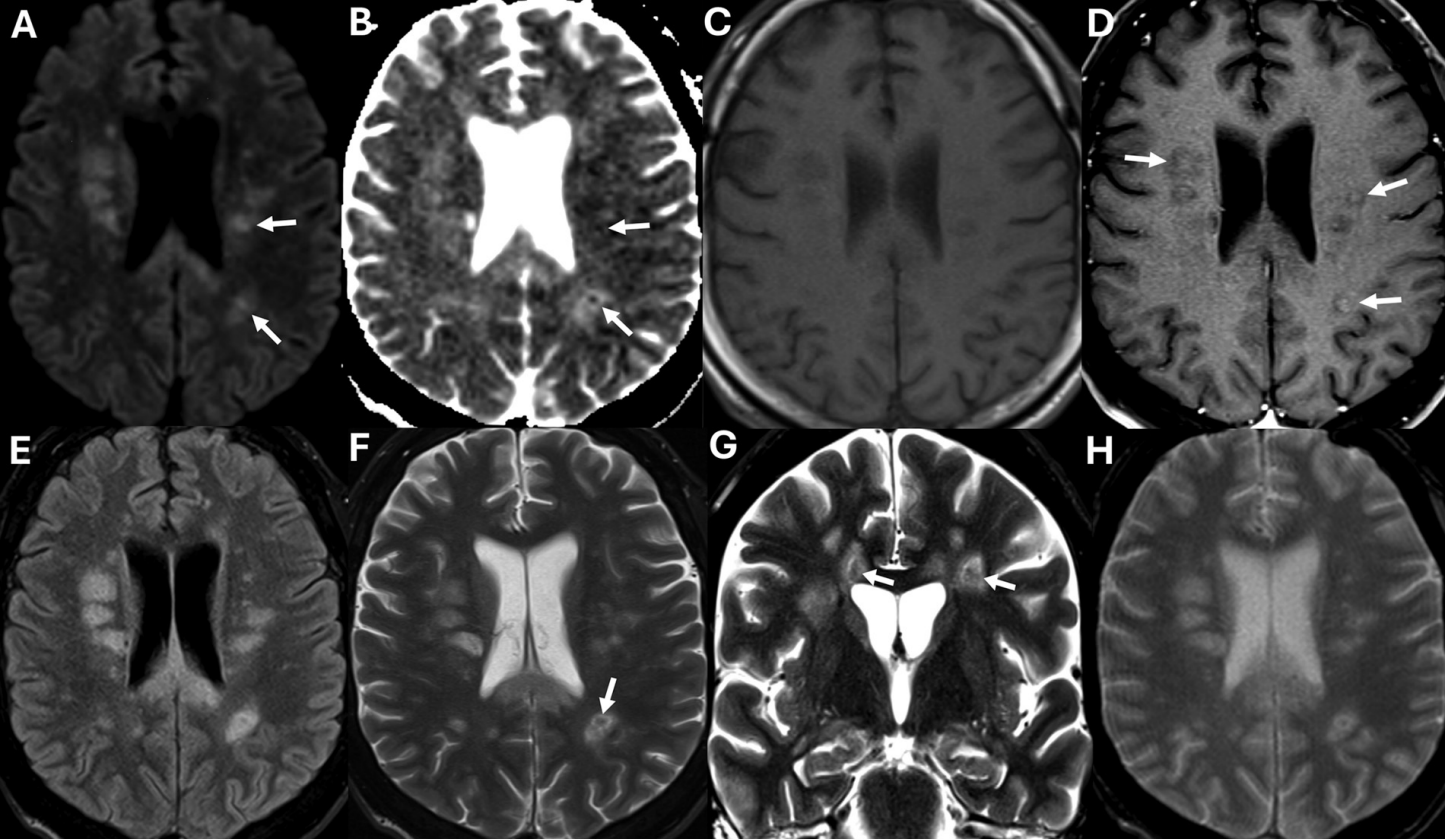
Initial brain MRI. ( **A,** **B** ) Axial diffusion-weighted imaging (DWI) and apparent diffusion coefficient (ADC) maps showing foci of high signal intensity with restricted diffusion in the deep white matter of both cerebral hemispheres and the corpus callosum. ( **C,** **D** ) Pre- and postcontrast T1-weighted showing mild contrast enhancement. ( **E,** **F** ) Axial fluid-attenuated inversion recovery (FLAIR)/T2 and ( **G** ) Coronal T2 showing heterogeneous hyperintensity within the lesions. ( **H** ) T2-weighted showing no significant magnetic susceptibility effect; however, given the lower sensitivity of T2 compared with susceptibility-weighted imaging (SWI), subtle hemorrhagic components – particularly in the lesion indicated by the arrow in panel F, which already presents low signal on T2–may have been present but undetected at this stage.

**Figure 2:**
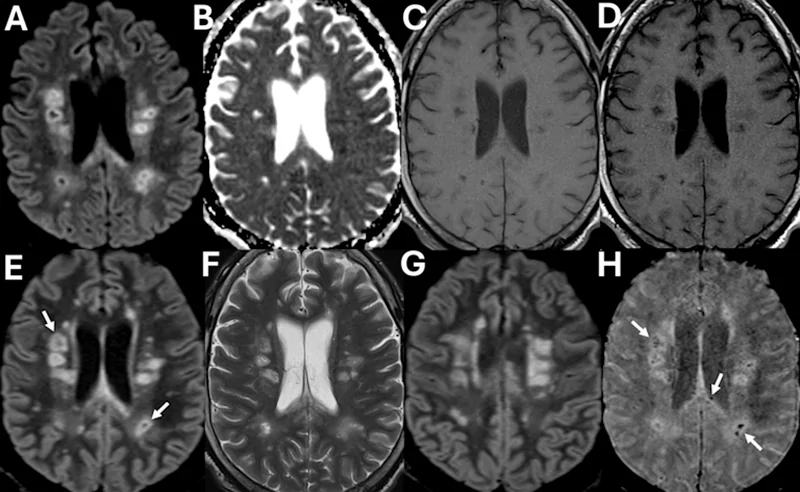
Follow-up brain MRI at 1 month. ( **A,** **B** ) Axial DWI and ADC maps showing no evidence of diffusion restriction. ( **C,** **D** ) Pre- and post-contrast T1 showing no enhancement. ( **E,** **F** ) Axial FLAIR/T2 showing some cavitation (arrows). ( **G** ) Axial FLAIR image at the level of the centrum semioval showing more confluent lesions. ( **H** ) Axial SWI showing multiple magnetic susceptibility artifacts in the centrum semioval and corpus callosum, consistent with microbleeds.

A 40-year-old man with metabolic syndrome developed acute respiratory failure after flu-like symptoms due to metapneumovirus infection. Despite pulmonary improvement, he remained unresponsive, with pyramidal signs. Brain magnetic resonance imaging (MRI) revealed multifocal T2/fluid-attenuated inversion recovery (FLAIR) hyperintensities involving white matter, brainstem, and splenium, consistent with acute disseminated encephalomyelitis (ADEM), as shown in Figure 1 . Cerebrospinal fluid (CSF) and electroencephalogram (EEG) were unremarkable. Minimal neurological recovery followed treatment with intravenous methylprednisolone and plasmapheresis. Follow-up MRI showed cavitation and hemosiderin deposits, suggesting viral-associated necrotizing acute disseminated leukoencephalopathy (VANDAL), 1 2 as shown in Figure 2 . This case expands the spectrum of VANDAL beyond COVID-19 and underscores its importance in the differential diagnosis of refractory ADEM secondary to metapneumovirus infection.
